# Interaction between ambient CO and temperature or relative humidity on the risk of stroke hospitalization

**DOI:** 10.1038/s41598-024-67568-8

**Published:** 2024-07-20

**Authors:** Zhuo Liu, Hua Meng, Xingtian Wang, Wenwen Lu, Xiaojuan Ma, Yuhui Geng, Xinya Su, Dongfeng Pan, Peifeng Liang

**Affiliations:** 1 School of Public Health and Management, Ningxia Medical University, Yinchuan, 750001 China; 2 Ningxia Key Laboratory of Environmental Factors and Chronic Disease Control, Yinchuan, 750000 China; 3 Department of Emergency Medicine, People’s Hospital of Ningxia Hui Autonomous Region, Yinchuan, 750000 China; 4 Public Health Center, People’s Hospital of Ningxia Hui Autonomous Region, Ningxia Medical University, 301 Zhengyuan North Street, Yinchuan, 750000 Ningxia China; 5 General hospital of Ningxia Medical University, No. 804, Shengli Street, Xingqing District, Yinchuan, 750001 Ningxia China; 6 Shenzhen Futian District Chronic Disease Prevention and Treatment Hospital, 18 Xinzhou 8Th Street, Futian District, Shenzhen, 518048 China

**Keywords:** Ambient CO, Meteorological factors, Stroke, Short-term effects, Interaction, Epidemiology, Environmental impact, Stroke

## Abstract

Although the independent effects of ambient CO, temperature or humidity on stroke have been confirmed, it is still unclear where there is an interaction between these factors and who is sensitive populations for these. The stroke hospitalization and ambient CO, temperature, humidity data were collected in 22 Counties and districts of Ningxia, China in 2014–2019. The lagged effect of ambient CO, temperature or humidity were analyze by the generalized additive model; the interaction were evaluated by the bivariate response surface model and stratified analysis with relative excessive risk (RERI). High temperature and CO levels had synergistic effects on hemorrhagic stroke (RERI = 0.05, 95% CI 0.033–0.086) and ischemic stroke (RERI = 0.035, 95% CI 0.006–0.08). Low relative humidity and CO were synergistic in hemorrhagic stroke (RERI = 0.192, 95% CI 0.184–0.205) and only in ischemic stroke in the elderly group (RERI = 0.056, 95% CI 0.025–0.085). High relative humidity and CO exhibited antagonistic effects on the risk of ischemic stroke hospitalization in both male and female groups (RERI = − 0.088, 95% CI − 0.151to − 0.031; RERI = − 0.144, 95% CI − 0.216 to − 0.197). Exposure to CO increases the risk of hospitalization related to hemorrhagic and ischemic strokes. CO and temperature or humidity interact with risk of stroke hospitalization with sex and age differences.

## Introduction

Experimental and clinical studies suggest that low concentrations of exogenous carbon monoxide (CO) may have beneficial neuroprotective effects in some cases^1–5^. However, population-based epidemiological studies of environmental CO exposure yielded mixed results. In a meta-analysis that involved 6.2 million events in 28 countries hospital admissions and deaths from stroke were associated with increased CO concentrations^6^. However, other multi-center studies showed that stroke admissions are either not or inversely correlated with ambient CO, after adjusting the concentrations for other air pollutants like SO_2_ and nitrogen dioxide^7–11^. This suggests that the health effects of environmental CO on stroke are still unclear, and the effect values vary based on other pollutants present.

The independent effects of atmospheric pollutants and meteorological factors on stroke have been supported by much evidence^12,13^. However, the risk factors for stroke are unlikely to occur alone but in clusters. Some scholars pointed out that air pollution will accelerate climate change^14^, which will, in turn, also change the emission and diffusion of pollutants. Therefore, the health risks caused by the combined action of air pollutants and meteorological factors may be greater than the single role of atmospheric pollutants or meteorological factors.

CO is one of the six major air pollutants and is generally produced by the incomplete combustion of fossil fuels such as motor vehicles^15–17^. Environmental factors, including temperature and humidity, can exert an impact on a car’s CO emissions. For example, in low-temperature, humid environments, the combustion efficiency of cars decreases, while the carbon monoxide emissions increase. In addition, when the car is driving at low speed in congested road conditions, the fuel combustion efficiency reduces, resulting in increased carbon monoxide emissions^18,19^. However, there is no clear evidence on whether temperature and humidity have a synergistic effect with environmental CO to increase the risk of stroke hospitalization in people.

In this study, we used time series analysis to examine the relationship between short-term exposure to environmental CO and the risk of stroke hospitalization. Considering that transportation is one of the major sources of carbon monoxide, we adjusted other transport-related pollutants such as PM2.5 or nitrogen dioxide. More importantly, we analyzed the effects of the interactions between temperature, humidity, and carbon monoxide on stroke hospitalizations in residents. We also performed subgroup analyses based on sex, age, and stroke subtypes to determine the subsets that are more susceptible to the effects of CO.

## Materials and methods

The ethics committee of People’s Hospital of Ningxia Hui Autonomous Region approved this study (Approval number: 2020-ZDYF-001). All methods were carried out in accordance with relevant guidelines and regulations. All participants and/or their legal guardians have informed consent. The datasets generated during and analyzed during the current study are available from the corresponding author on reasonable request.

### Study area

This study was implemented in the Ningxia, which located in northwest China and has jurisdiction over five prefecture-level cities 22 Counties and districts with a total area of 66,400 km^2^ and a permanent resident population of 7.25 million. The average temperature in Ningxia between 5.6 and 10.1 °C, and the annual precipitation between 167.2 and 674.3 mm.

### Hospitalization data

The records of stroke were collected from the homepage of the discharge medical records of 56 hospitals in 22 Counties and districts of Ningxia. The daily hospitalization data of stroke, including admission date, age, gender, current address, and discharge diagnosis were screened from January 1, 2015, to December 31, 2019. Based on the incidence characteristics of stroke, the hospitalized cases of stroke were classified into either hemorrhagic (ICD-10: I60–I61) or ischemic stroke (ICD-10: I62–I63).The study excluded individuals under 18 years of age and non-local residences of residence for less than 6 months. Considering the differences in sensitivity to pollutants and meteorological factors among people of different ages and genders, the study divided hospitalized stroke cases into two subgroups, which are, adult (18–64 years old) and the elderly (≥ 65 years old), as well as two subgroups of males and females.

The ethics committee of People’s Hospital of Ningxia Hui Autonomous Region approved this study (Approval number: 2020-ZDYF-001). The datasets generated during and analysed during the current study are available from the corresponding author on reasonable request.All methods were carried out in accordance with relevant guidelines and regulations.All participants and/or their legal guardians have informed consent.

### Air pollution and meteorological data

Atmospheric pollution data were obtained from the National Urban Air Quality Real-Time Dissemination Platform (https://air.cnemc. cn:18007/), which is operated by the National Environmental Monitoring Center of China, in accordance with China’s Ambient Air Quality Standards (GB3095-2012). The simultaneous meteorological data were obtained from the China Meteorological Data Service Center (http://data.cma.cn/). Data on air pollutants in the Ningxia region were obtained by summarizing the data from five municipalities, as well as the arithmetic averages: the 24-h average concentration of CO (mg/m^3^), daily average temperature (°C), and the daily average relative humidity (%) over the patient’s region.

### Generalized additive model (GAM)

A time-series approach was used to evaluate the short-term effects of CO on the risk of stroke-related hospitalization. Considering that the number of daily hospitalization cases related to stroke is a small probability event relative to the resident population in Ningxia, and the distribution type is similar to Poisson, the study used a Generalized Additive Model (GAM) with Poisson-like regression to incorporate CO into a single-pollutant model. In light of the lagged nature of the effects of CO on health, the study analyzed the one-day lagged effects on the same day of CO exposure and from days 1 to 7 (Lag1–Lag7) after exposure were analyzed. The cumulative lagged effects were also analyzed by using the moving average of the concentrations from days 1 to 7 after CO exposure (Lag01–Lag07). The study used generalized cross-validation (GCV) to control confounding factors such as time trends, temperature, and relative humidity. Day-of-the-week and vacation effects were adjusted by dummy variables in the model. The degrees of freedom of the model were estimated based on the GCV and were set to seven per year for the time trend and three for temperature and relative humidity.

The GAM formula is as follows:

1 $$Y_{t} \sim quasipoisson \left( {\mu_{t} } \right)$$

2 $$\begin{aligned} \log \left( {\mu_{t} } \right) = & \beta_{0} + \beta_{1} X_{t} + ns\left( {time, df} \right) + ns\left( {Z_{t} , df} \right) \\ & \; + factor\left( {DOW_{t} } \right) + factor \left( {holiday_{t} } \right) \\ \end{aligned}$$

In the above equation: $Y_{t}$—number of stroke hospitalizations on day t; $\mu_{t}$—expected number of stroke hospitalizations at day t; ${\upbeta }_{0}$—intercept; ${\upbeta }_{1} {\text{X}}_{{\text{t}}} {-\!\!-}{\upbeta }_{1}$ is the regression coefficient of the effect of air pollutants on stroke hospitalization, and is the value of air pollutant concentration on day t; ${\text{ns}}$—natural cubic spline functions controlling for confounding factors such as time trends, temperature, relative humidity, etc.; the ${\text{time}}$—time-variant, ${\text{time}} = 1,{ }2,{ }3 \ldots 1826$; df—degree of freedom; ${\text{Z}}_{{\text{t}}}$—meteorological factor variables on day t, including air temperature, relative humidity; ${\text{DOW}}_{{\text{t}}}$—a dichotomous variable for the weekend effect, with weekdays as “0” and weekends as “1”;$holiday_{t}$—a dichotomous variable for the vacation effect, with “0” for weekdays and “1” for vacations.

Based on the regression coefficients of CO and its standard error (SE) estimated by the model, the percentage changes in the risk of stroke-related hospitalization for each unit increase in CO concentration (per increase in CO) was calculated as the excess risk (excessive risk, ER): ER = 0 indicated that the risk of morbidity was the same in both the exposed and control groups; ER < 0, which indicated that the risk of morbidity in the exposed group was lower than that in the control group; ER > 0 means that the risk of morbidity in the exposed group is higher than that in the control group.

### Sensitivity analysis

#### Dual-pollutant model

After using the single-pollutant model for estimating the health effects of CO, a two-pollutant model was further constructed by introducing the other air pollutants (PM_2.5_, PM_10_, SO_2_, NO_2_ and O_3_) separately and in a linear form. The number of lagged days that were meaningful, with the largest effect values in the one-factor analysis, were considered. Observing the change in effect values can be used to verify the stability of the model, in addition to testing whether the health-related effects of one pollutant are affected by other co-existing pollutants. The dual-pollutant model equation is as follows:

3 $$Y_{t} \sim Poisson \left( {\mu_{t} } \right)$$

4 $$\begin{aligned} \log \left( {\mu_{t} } \right) = & \beta_{0} + \beta_{1} X_{t} + \beta_{2} X_{t0} + ns\left( {time, df} \right) + ns\left( {Z_{t} , df} \right) \\ & \; + factor\left( {DOW_{t} } \right) + factor \left( {holiday_{t} } \right) \\ \end{aligned}$$

${\upbeta }_{2} {\text{X}}_{{{\text{t}}0}}$—${\upbeta }_{2}$ are regression coefficients for the effect on hospitalization for hemorrhagic stroke after adjusting for air pollutants. ${\text{X}}_{{{\text{t}}0}}$ is the concentration value of another air pollutant on day t. Other variables are the same as Eq. (2).

#### Freedom to change

The stability of the main model that was constructed in this study was verified by changing the degrees of freedom of the time trend and other confounding factors in the single-pollutant model. The changes in the effect values were then observed after the alterations in the parameters.

#### Exposure-response curves

Finally, the exposure-response relationship curves between environmental CO and the risk of hospitalization in the population were plotted, based on the GAM.

### Bivariate response surface model

Due to the varying nature and units of CO as well as temperature and humidity, the study used the tensor product smoothing function (Te) to build a bivariate response surface model of these parameters. The response planes of CO-temperature and humidity-health outcomes were plotted to qualitatively determine possible interaction between the two.

5 $$\begin{aligned} Log{\text{E}}Y_{t} = & {\upalpha } + {\text{Te}}\left( {Pol_{t} ,Met_{t} } \right) + ns\left( {Z_{t} ,df} \right) + ns\left( {time,df} \right) \\ & \; + factor\left( {DOW_{t} } \right) + factor \left( {holiday_{t} } \right) \\ \end{aligned}$$

Tensor product smoothing function for the interaction of Te–CO with air temperature and humidity, ${\text{Pol}}_{{\text{t}}}$ is the concentration of the pollutant on day t, ${\text{Met}}_{{\text{t}}}$ denotes the value of the meteorological factor on day t.

### Interaction analysis

The study used the median ambient CO concentration (*P*_50_) as the cut-off point to classify CO into low and high levels. The* P*_5_ and *P*_95_ of air temperature and humidity were used as cut-off points to categorize these parameters into low, medium, and high levels. A two-by-two combination of ambient CO concentration levels and temperature and humidity levels were performed to analyze the effects of the interaction between temperature, humidity, and ambient CO on the risk of stroke-related hospitalization. The combination of the medium levels of temperature and humidity- and the low concentration of CO was used as a reference to this effect. The direction and magnitude of the interaction were assessed using the relative excessive risk due to interaction (*RERI*):

8 $$RERI = R_{11} - R_{01} - R_{10} + R_{00}$$

*R*_11_ is the *RR* value when exposed to both low and high levels of temperature and humidity, as well as high levels of CO pollution. *R*_10_ and *R*_01_ are the *RR* values when exposed to low or high levels of temperature and humidity, as well as low levels of CO pollution. Alternatively, these might be *RR* values when exposed to medium levels of temperature and humidity as well as high levels of CO pollution. *R*_00_ is the *RR* value when exposed to medium levels of temperature and humidity as well as low levels of CO pollution, which is set to be 1. When the *RERI* > 0 and the 95% CI does not contain 0, a synergistic interaction exists between the two factors. When *RERI* < 0 and the 95% CI does not contain 0, an antagonistic interaction exists between the two factors. When *RERI* = 0, no interaction exists.

### Statistical analysis

Excel 2019 was used to clean and organize the raw data while descriptive analysis was done using SPSS25.0. The main statistical analysis was carried out using R4.1.2 software. The “splines” package was used to call the natural cubic spline function. Both the generalized summation model and the generalized additive model were constructed using the “mgcv” package. The test level was α = 0.05, and a two-sided *P* value of < 0.05 was considered statistically significant.

### Ethical approval

The ethics review committee of The People’s Hospital of Ningxia Hui Autonomous Region approved this study (Approval number: 2020-KY-053). The authors have reported that they have no relationships relevant to the contents of this paper to disclose.

## Results

### Basic information

A total of 138,851 stroke cases were included in the study, of which 121,946 (87.83%) were ischemic strokes and 16,905 (12.17%) were hemorrhagic strokes. In the age subgroup, the results were as follows: 84,528 (69.3%) ischemic strokes in young people and 17,418 (30.7%) in the elderly; 8752 (51.8%) hemorrhagic strokes in young people and 8153 (48.2%) in the elderly. In the gender subgroup, the findings for ischemic stroke showed that there were 63,698 (52.2%) and 58,248 (47.8%) in males and females, respectively. In the gender subgroup, there were 10,244 (60.6%) male and 6661 (39.4%) female cases of ischemic stroke and hemorrhagic stroke.

**Figure 1:**
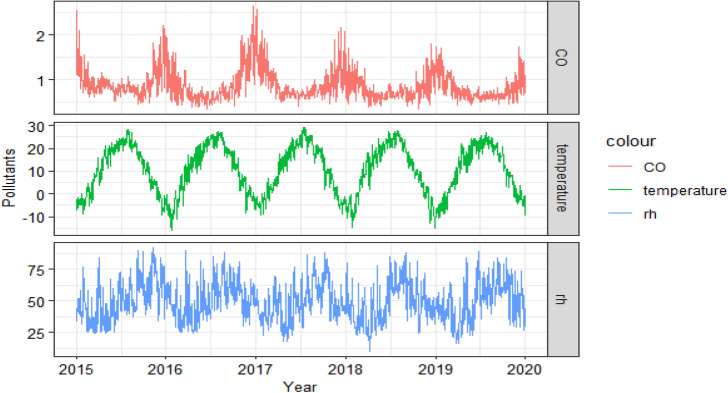
Time series plot of CO concentration versus meteorological factors in Ningxia, 2015–2019.

During the study period, the average daily concentration of CO in Ningxia was 0.87 mg/m^3^, which was lower than the limit of the secondary standard in the Ambient Air Quality Standard (GB 3095-2012). The average temperature in spring is 12.42 °C, the average temperature in summer is 22.91 °C, the average temperature in autumn is 10.52 °C, and the average temperature in winter is − 3.68 °C. The average relative humidity in spring was 38.33%, 54.16% in summer, 58.47% in autumn and 45.37% in winter. As shown in the time series plot (Fig. 1), the CO concentration was relatively higher in winter and spring, and it reduced in summer and fall. Apparently, the average daily temperatures and relative humidity showed a cyclical trend, with the former being higher in summer than in winter and the latter being higher in summer and fall, compared to the winter and spring.

### Lagged effect of CO on the risk of stroke hospitalization

**Figure 2:**
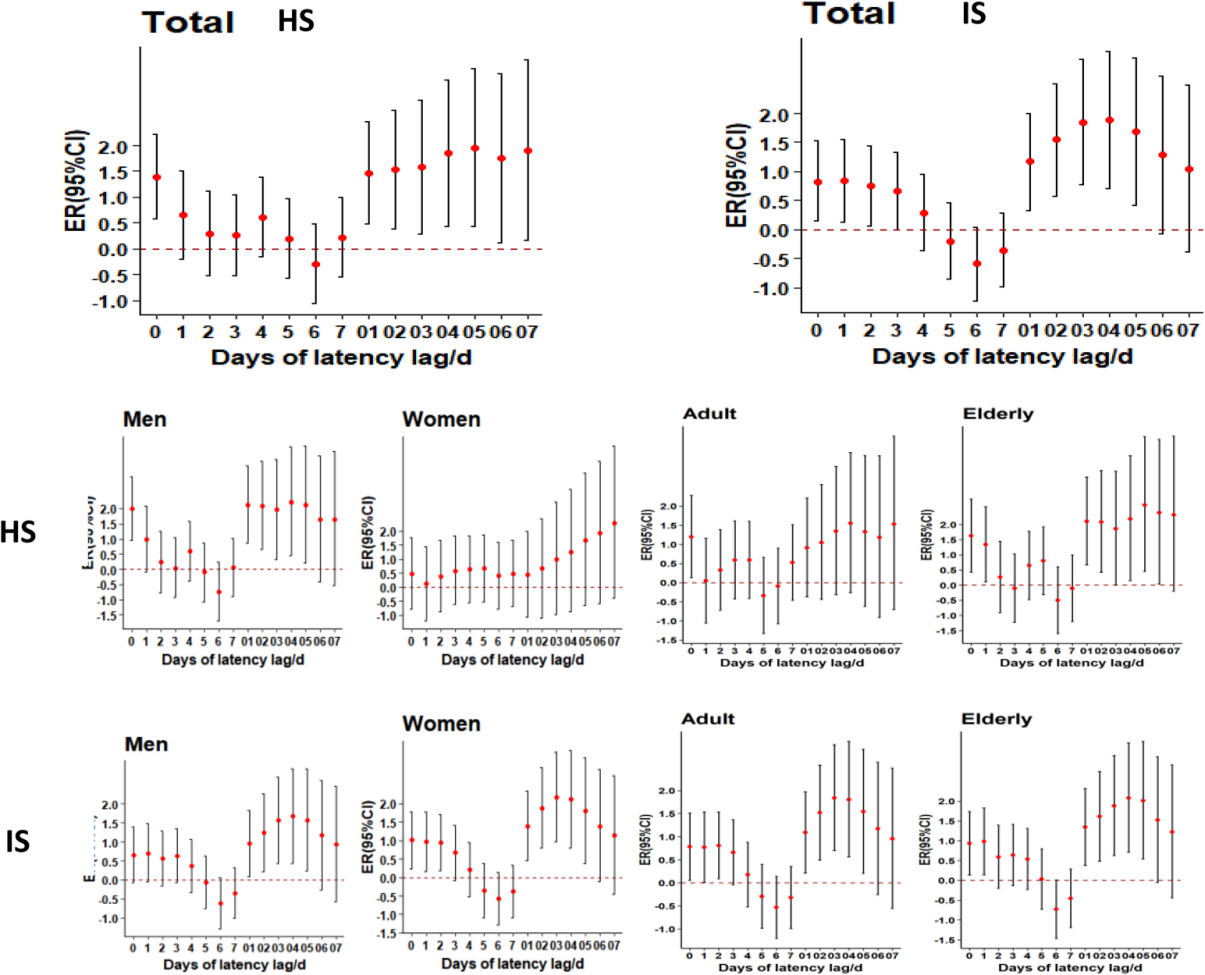
Plot of lagged effect of each 0.1 mg/m^3^ increase in ambient CO on the risk of hospitalization for hemorrhagic stroke [ER (95% CI)].

The lagged effect of CO on the risk of hospitalization for hemorrhagic stroke is shown in Table 1 and Fig. 2. The single-day lagged and cumulative lagged effects of CO on the risk of hospitalization for the total population of hemorrhagic stroke were found at Lag0 (*ER* = 1.394, 95% CI 0.573–2.221), and Lag05 (*ER* = 1.945, 95% CI 0.432–3.480), respectively. The largest effect value was observed. In the age subgroups, the single-day lagged effect of CO on hospitalization in the young and middle-aged group with hemorrhagic stroke was at Lag0 (*ER* = 1.197, 95% CI 0.136–2.268) on the day of exposure. The single-day lagged and cumulative lagged effects of CO on the risk of hospitalization in the elderly group with hemorrhagic stroke were at Lag0 (*ER* = 1.625, 95% CI 0.436–2.829) and Lag05 (*ER* = 2.646, 95% CI 0.448–4.893), respectively, with the largest effect values being noted. For the gender subgroups, the one-day lagged effect of CO on the risk of hospitalization in the male participants with hemorrhagic stroke was found at Lag0 (*ER* = 1.987, 95% CI 0.954–3.030), while the cumulative lagged effect was at Lag04 (*ER* = 2.220, 95% CI 0.453–4.019), with the largest effect values being noted. The effects of CO on the risk of hospitalization in the female group of participants with hemorrhagic stroke were not statistically significant (*P* > 0.05).

**Table 1:** Effect of each 0.1 mg/m^3^ increase in ambient CO on stroke hospitalization in the population [ER (95%*CI*)].

	CO	Total population	Adult	Elderly	Male	Female
Hemorrhagic stroke						
Single-day lag effect	Lag0	**1.394 (0.573**–2.221)*	**1.197 (0.136**–2.268)*	**1.625 (0.436**–2.829)*	**1.987 (0.954**–3.030)*	0.490 (− 0.770 to 1.767)
	Lag1	0.655 (− 0.196 to 1.514)	0.046 (− 1.053 to 1.156)	**1.342 (0.105**–2.593)*	0.997 (− 0.076 to 2.081)	0.124 (− 1.186 to 1.452)
	Lag2	0.295 (− 0.513 to 1.110)	0.326 (− 0.720 to 1.384)	0.261 (− 0.909 to 1.444)	0.228 (− 0.785 to 1.252)	0.393 (− 0.861 to 1.663)
	Lag3	0.260 (− 0.523 to 1.049)	0.590 (− 0.420 to 1.616)	− 0.104 (− 1.234 to 1.039)	0.045 (− 0.936 to 1.036)	0.591 (− 0.624 to 1.820)
	Lag4	0.614 (− 0.161 to 1.375)	0.588 (− 0.416 to 1.602)	0.645 (− 0.476 to 1.779)	0.603 (0.370–1.586)*	0.631 (− 0.568 to 1.845)
	Lag5	0.202 (− 0.567 to 0.976)	− 0.340 (− 1.332 to 0.662)	0.800 (− 0.316 to 1.928)	− 0.101 (− 1.065 to 0.872)	0.674 (− 0.519 to 1.880)
	Lag6	− 0.284 (− 1.044 to 0.482)	− 0.093 (− 1.078 to 0.902)	− 0.502 (− 1.598 to 0.606)	− 0.735 (− 1.686 to 0.226)	0.419 (− 0.762 to 1.615)
	Lag7	0.226 (− 0.535 to 0.993)	0.523 (− 0.464 to 1.519)	− 0.109 (− 1.207 to 1.001)	0.055 (− 0.900 to 1.020)	0.451 (− 0.687 to 1.686)
Cumulative lag effect	Lag01	1.465 (0.476–2.464)*	0.908 (− 0.371 to 2.203)	2.100 (0.664–3.557)*	2.129 (0.880–3.394)*	0.493 (− 1.066 to 1.991)
	Lag02	**1.532 (0.384**–2.691)*	1.048 (− 0.434 to 2.552)	**2.081 (0.421**–3.773)*	**2.095 (0.649**–3.563)*	0.665 (− 1.095 to 2.457)
	Lag03	**1.583 (0.302**–2.881)*	1.345 (− 0.313 to 3.031)	1.863 (− 0.007 to 3.753)	**1.959 (0.344**–3.601)*	1.001 (− 0.970 to 3.010)
	Lag04	**1.845 (0.444**–3.266)*	1.550 (− 0.261 to 3.393)	**2.188 (0.157**–4.261)*	**2.220 (0.453**–4.019)*	1.265 (− 0.888 to 3.465)
	Lag05	**1.945 (0.432**–3.480)*	1.328 (− 0.621 to 3.314)	**2.646 (0.448**–4.893)*	**2.126 (0.220**–4.068)*	1.664 (− 0.662 to 4.045)
	Lag06	**1.749 (0.126**–3.398)*	1.180 (− 0.909 to 3.313)	**2.293 (0.036**–4.800)*	1.635 (− 0.406 to 3.718)	1.921 (− 0.580 to 4.485)
	Lag07	**1.904 (0.166**–3.672)*	1.531 (− 0.707 to 3.820)	2.323 (− 0.196 to 4.905)	1.650 (− 0.534 to 3.882)	2.289 (− 0.389 to 5.039)
Ischemic stroke						
Single-day lag effect	Lag0	0.829 (0.141–1.522)*	0.783 (0.057–1.515)*	0.933 (0.133–1.739)*	0.661 (− 0.064 to 1.392)	1.011 (0.244–1.784)*
	Lag1	0.837 (0.116–1.563)*	0.773 (0.012–1.541)*	0.982 (0.145–1.826)*	0.709 (− 0.051 to 1.475)	0.975 (0.171–1.785)*
	Lag2	0.743 (0.058–1.432)*	0.809 (0.085–1.538)*	0.591 (− 0.201 to 1.391)	0.562 (− 0.159 to 1.291)	0.938 (0.174–1.708)*
	Lag3	0.653 (− 0.011 to 1.322)	0.659 (− 0.043 to 1.367)	0.636 (− 0.133 to 1.413)	0.643 (− 0.058 to 1.349)	0.663 (− 0.078 to 1.411)
	Lag4	0.285 (− 0.373 to 0.948)	0.174 (− 0.521 to 0.875)	0.537 (− 0.226 to 1.308)	0.363 (− 0.332 to 1.064)	0.199 (− 0.534 to 0.938)
	Lag5	− 0.196 (− 0.849 to 0.461)	− 0.297 (− 0.987 to 0.397)	0.031 (− 0.727 to 0.796)	− 0.056 (− 0.747 to 0.639)	− 0.349 (− 1.075 to 0.382)
	Lag6	− 0.591 (− 1.226 to 0.046)*	− 0.533 (− 1.204 to 0.141)	− 0.729 (− 1.465 to 0.012)	− 0.613 (− 1.284 to 0.061)	− 0.567 (− 1.272 to 0.143)
	Lag7	− 0.361 (− 0.994 to 0.276)	− 0.3204 (− 0.989 to 0.353)	− 0.456 (− 1.191 to 0.284)	− 0.346 (− 1.016 to 0.328)	− 0.375 (− 1.079 to 0.333)
Single-day lag effect	Lag01	1.165 (0.332–2.006)*	1.089 (0.209–1.976)*	1.342 (0.373–2.321)*	0.957 (0.078–1.844)*	1.392 (0.463–2.3303)*
	Lag02	1.546 (0.579–2.523)*	1.518 (0.496–2.5502)*	1.611 (0.487–2.748)*	1.243 (0.222–2.273)*	1.876 (0.796–2.968)*
	Lag03	1.854 (0.773–2.946)*	1.841 (0.7001–2.994)*	1.883 (0.628–3.154)*	1.566 (0.427–2.718)*	2.166 (0.96–3.387)*
	Lag04	1.889 (0.712–3.081)*	1.805 (0.562–3.064)*	2.083 (0.713–3.471)*	1.68 (0.436–2.939)*	2.116 (0.803–3.446)*
	Lag05	1.684 (0.418–2.967)*	1.541 (0.205–2.895)*	2.012 (0.538–3.508)*	1.575 (0.236–2.932)*	1.8008 (0.391–3.231)*
	Lag06	1.278 (− 0.0703 to 2.645)	1.171 (− 0.252 to 2.614)	**1.522 (**− 0.046 to 3.116)*	1.171 (− 0.255 to 2.618)	1.392 (− 0.106 to 2.914)
	Lag07	1.036 (− 0.396 to 2.489)	0.953 (− 0.558 to 2.487)	1.223 (− 0.442 to 2.917)	0.937 (− 0.579 to 2.477)	1.142 (− 0.448 to 2.758)

Significant values are in bold.

* indicates P < 0.05.

The results for the lagged effect of CO on the risk of hospitalization for ischemic stroke are presented in Table 1 and Fig. 2. The single-day and cumulative lagged effects of CO on the risk of hospitalization for the whole ischemic stroke population were at Lag1 (*ER* = 0.837, 95% CI 0.116–1.563) and Lag04 (*ER* = 1.889, 95% CI 0.712–3.081), respectively, with the effect values being the largest. In the age subgroups, the largest effect values for the single-day and cumulative lagged effects of CO on the risk of hospitalization in the young and middle-aged group of participants with ischemic stroke were found at Lag3 (*ER* = 0.809, 95% CI 0.085–1.538) and Lag03 (*ER* = 1.841, 95% CI 0.7001–2.994), respectively. The single-day lagged and cumulative lagged effects of CO on the risk of hospitalization in the old and middle-aged groups of ischemic stroke were the largest at Lag3 (*ER* = 0.891, 95% CI 0.7001–2.994). Apparently, the single-day and cumulative lagged effects were largest at Lag1 (*ER* = 0.982, 95% CI 0.145–1.826) and Lag04 (*ER* = 2.083, 95% CI 0.713–3.471), respectively. In the gender subgroup, no statistical significance (*P* > 0.05) was noted for the single-day lagged effect of CO on the risk of hospitalization in the male participants with ischemic stroke. However, the cumulative lagged effect had the largest effect value at Lag04 (*ER* = 1.68, 95% CI 0.436–2.939). In the female group of ischemic stroke, the one-day and cumulative lagged effects of CO on the risk of hospitalization were statistically significant at Lag0 (*ER* = 1.011, 95% CI 0.244–1.784) and Lag03 (*ER* = 2.166, 95% CI 0.96–3.387), where the effect values were the largest.

### Sensitivity analysis

Based on the number of lag days with the strongest pollutant effects, sensitivity analyses were done by both constructing a two-pollutant model and changing the degrees of freedom in a bid to judge the stability of the model. In the two-pollutant model, the other pollutants were analyzed separately by including them in the GAM in a linear form (Table 2). The results of the two-pollutant model remained essentially stable, except for a statistically insignificant number of days of lag between CO and the risk of stroke hospitalization, after adjusting for NO_2_ and PM2.5.

**Table 2:** Sensitivity analysis results.

	HS (Lag0)	HS (Lag05)	IS (Lag1)	IS (Lag04)
Master model results	1.394 (0.573–2.221)*	1.945 (0.432–3.480)*	0.837 (0.116–1.563)*	1.889 (0.712–3.081)*
Dual contamination model				
Adjustment of PM2.5	**1.008 (0.04**–1.987)*	1.495 (− 0.047 to 3.063)	**.0.875 (0.111**–1.645)*	**1.973 (0.744**–3.217)*
Adjustment of PM_10_	**1.361 (0.539**–2.189)*	**1.851 (0.329**–3.397)*	**0.908 (0.175**–1.646)*	**1.987 (0.798**–3.191)*
Adjustment of SO_2_	**1.81 (0.582**–3.053)*	**1.784 (0.259**–3.331)*	**0.801 (0.059**–1.548)*	**1.859 (0.658**–3.073)*
Adjustment of NO_2_	0.786 (− 0.562 to 2.153)	1.460 (− 0.079 to 3.023)	0.684 (− 0.061 to 1.435)	**1.672 (0.451**–2.908)*
Adjustment of O_3_	**1.338 (0.511**–2.171)*	**1.842 (0.322**–3.386)*	**0.858 (0.136**–1.586)*	**1.969 (0.784**–3.168)*
Change the degrees of freedom				
*df*_time_ = 6	**1.351 (0.532**–2.176)*	**1.843 (0.343**–3.363)*	**0.719 (**− **0.014** to 1.458)*	**1.555 (0.363**–2.761)*
*df*_time_ = 8	**1.372 (0.551**–2.201)*	**2.001 (0.480**–3.544)*	**0.763 (0.049**–1.481)*	**1.854 (0.681**–3.041)*
*df*_time_ = 9	**1.331 (0.508**–2.161)*	**1.848 (0.318**–3.401)*	**0.838 (0.126**–1.555)*	**2.137 (0.958**–3.328)*
*df*_time_ = 10	**1.308 (0.486**–2.137)*	**1.803 (0.268**–3.361)*	**0.855 (0.143**–1.571)*	**2.222 (1.036**–3.422)*
*df*_temp_ = 4	**1.374 (0.553**–2.202)*	**1.910 (0.398**–3.444)*	**0.847 (0.127**–1.572)*	**1.888 (0.713**–3.078)*
*df*_temp_ = 5	**1.393 (0.573**–2.221)*	**2.023 (0.509**–3.559)*	**0.852 (0.131**–1.578)*	**1.918 (0.738**–3.111)*
*df*_rh_ = 4	**1.372 (0.5503**–2.201)*	**1.913 (0.399**–3.449)*	**0.839 (0.119**–1.564)*	**1.879 (0.702**–3.069)*
*df*_rh_ = 5	**1.361 (0.539**–2.191)*	**1.911 (0.398**–3.447)*	**0.844 (0.124**–1.569)*	**1.882 (0.706**–3.071)*

Significant values are in bold.

*indicates p < 0.05; dftime: degrees of freedom for fitting time trends; dftemp: degrees of freedom for fitting air temperature; dfrh: degrees of freedom for fitting relative humidity.

Compared with the results of the main model, changing the degrees of freedom of 6, 7, 9, and 10 for the time trend in the model, as well as 4 and 5 for temperature, relative humidity, and barometric pressure, respectively, did not affect the effect values.

**Figure 3:**
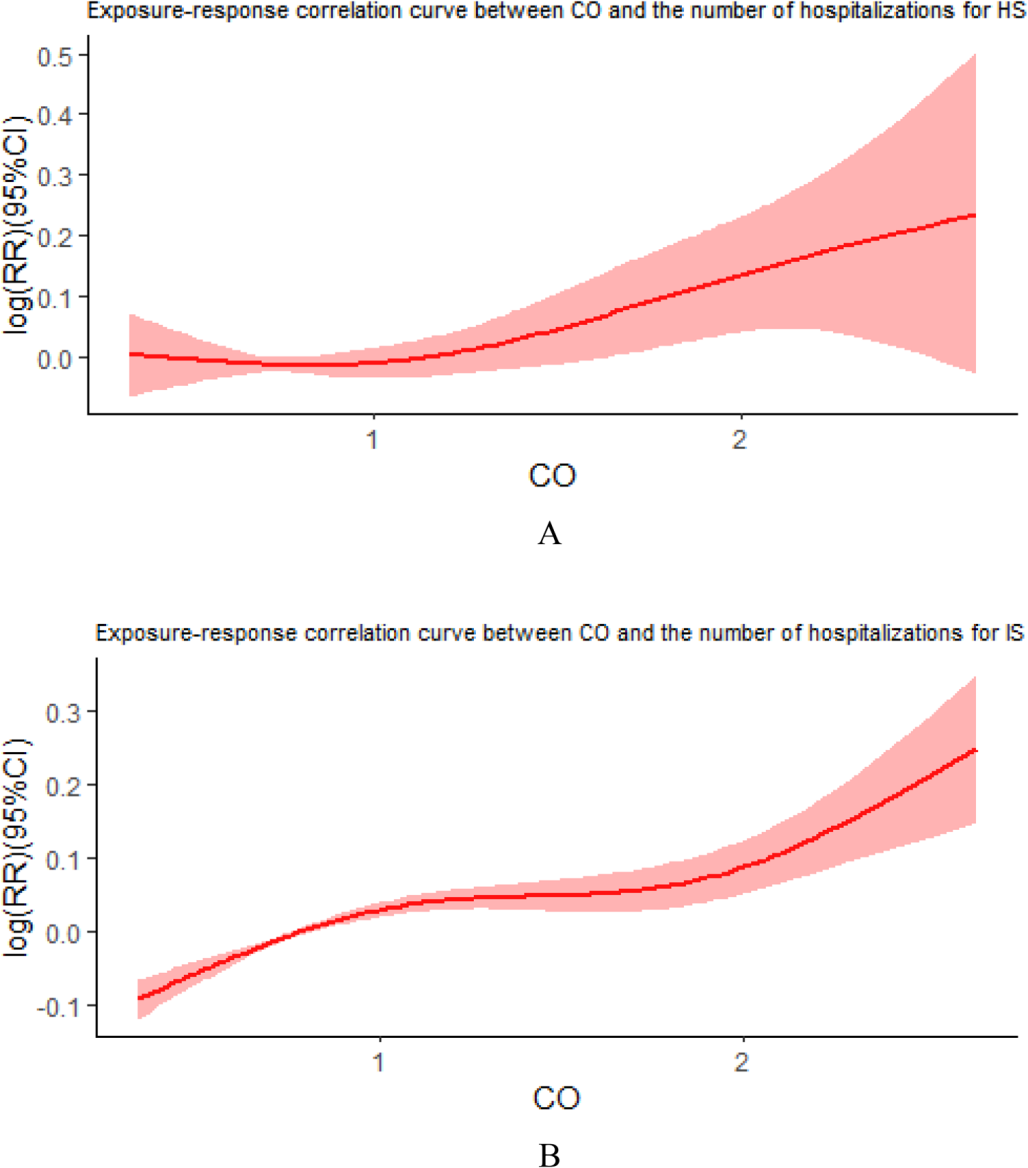
Exposure–response curves for the relationship between environmental CO and the risk of hospitalization for stroke in the population. (Figure labels **A** is the exposure-response relationship curve between CO and the number of hospitalizations for hemorrhagic stroke. Figure labels **B** is the exposure-response relationship curve between CO and the number of hospitalizations for ischemic stroke.)

The exposure-response relationship curves between ambient CO and residential stroke hospitalization risk (Fig. 3) show that there is a curvilinear positive trend between the two parameters when other pollutants are being controlled. The results indicate that the association between ambient CO and residential hospitalization risk obtained from the main model is stable and robust.

### Interaction of CO and temperature on risk of stroke hospitalization

**Figure 4:**
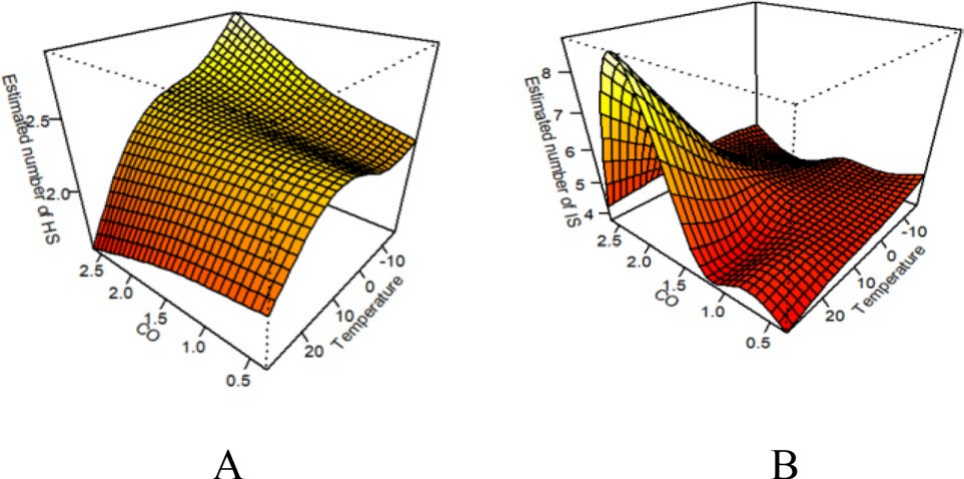
3D plot of the effect of ambient CO versus temperature on stroke. (**A **is a 3D plot of the effect of CO and temperature on hemorrhagic stroke. **B** is a 3D plot of the effect of CO and temperature on ischemic stroke.)

As shown in Fig. 4A and Table 3, high temperature and ambient CO had synergistic effects on the risk of hospitalization in the total population for hemorrhagic stroke (*RERI* = 0.05, 95% CI 0.033–0.086). Based on subgroup analyses, high temperature and CO had significant effects on the risk of hospitalization in the male group (*RERI* = 0.038, 95% CI 0.02–0.087), female group (*RERI* = 0.067, 95% CI 0.045–0.134), young and middle-aged group (*RERI* = 0.042, 95% CI 0.023–0.095), and elderly group (*RERI* = 0.058, 95% CI 0.038–0.117). Apparently, hypothermia and CO had no interaction with hemorrhagic stroke.

**Table 3:** Interaction between CO and meteorological factors on risk of stroke hospitalization.

Meteorological factors		CO level	Total population	Adult	Elderly	Male	Female
Hemorrhagic stroke							
Temperature	Medium	Low	Reference layer	Reference layer	Reference layer	Reference layer	Reference layer
		High	0.992 (0.946–1.041)	1.001 (0.941–1.066)	0.983 (0.918–1.052)	0.982 (0.925–1.043)	1.007 (0.936–1.085)
	Low	Low	1.003 (0.858–1.172)	1.052 (0.861–1.286)	0.9501 (0.756–1.193)	1.036 (0.855–1.255)	0.954 (0.741–1.227)
		High	1.033 (0.924–1.155)	1.035 (0.895–1.198)	1.031 (0.88–1.2101)	1.1104 (0.968–1.273)	0.914 (0.763–1.095)
		RERI^a^	0.038 (− 0.058 to 0.12)	− 0.018 (− 0.154 to 0.093)	0.097 (− 0.035 to 0.206)	0.092 (− 0.025 to 0.188)	− 0.047 (− 0.217 to 0.086)
	High	Low	0.893 (0.793–1.007)*	0.913 (0.782–1.066)	0.874 (0.736–1.038)	0.908 (0.781–1.055)	0.872 (0.726–1.048)
		High	0.938 (0.774–1.137)	0.955 (0.744–1.226)	0.921 (0.698–1.214)	0.9305 (0.728–1.188)	0.9505 (0.7104–1.271)
		RERI^b^	**0.05 (0.033**–0.086)*	**0.042 (0.023**–0.095)*	**0.058 (0.038**–0.117)*	**0.038 (0.02**–0.087)*	**0.067 (0.045**–0.134)*
Relatively humidity	Medium	Low	Reference layer	Reference layer	Reference layer	Reference layer	Reference layer
		High	0.995 (0.949–1.043)	0.985 (0.926–1.047)	1.006 (0.941–1.076)	0.995 (0.938–1.055)	0.995 (0.925–1.071)
	Low	Low	1.014 (0.908–1.132)	1.008 (0.871–1.167)	1.0203 (0.872–1.193)	0.941 (0.816–1.085)	1.128 (0.957–1.329)
		High	1.201 (1.062–1.359)	1.189 (1.011–1.399)	1.214 (1.018–1.447)	1.111 (0.948–1.303)	1.343 (1.116–1.616)
		RERI^a^	**0.192 (0.184**–0.205)*	**0.196 (0.185**–0.214)*	**0.1877 (0.178**–0.205)*	**0.175 (0.163**–0.194)*	**0.22 (0.216**–0.234)*
	High	Low	1.0005 (0.873–1.145)	1.037 (0.868–1.238)	0.963 (0.794–1.168)	1.066 (0.904–1.256)	0.896 (0.719–1.117)
		High	0.917 (0.814–1.033)	0.939 (0.805–1.095)	0.893 (0.753–1.061)	0.967 (0.835–1.121)	0.846 (0.702–1.021)
		RERI^b^	− **0.071 (**− **0.147 to **− 0.001)*	− 0.077 (− 0.185 to 0.016)	− 0.066 (− 0.173 to 0.028)	− 0.082 (− 0.178 to 0.005)	− 0.045 (− 0.167 to 0.058)
Ischemic stroke							
Temperature	Medium	Low	Reference layer	Reference layer	Reference layer	Reference layer	Reference layer
		High	1.02 (0.978–1.063)	1.013 (0.969–1.059)	1.035 (0.987–1.086)	1.018 (0.974–1.063)	1.022 (0.975–1.071)
	Low	Low	1.008 (0.884–1.148)	0.951 (0.826–1.095)	1.139 (0.986–1.316)	1.051 (0.918–1.202)	0.9603 (0.827–1.115)
		High	0.987 (0.896–1.087)	0.969 (0.874–1.073)	1.031 (0.922–1.152)	0.969 (0.875–1.073)	1.008 (0.904–1.123)
	RERI^a^		− 0.041 (− 0.124 to 0.034)	0.005 (− 0.081 to 0.079)	− **0.143 (**− **0.25 to **− 0.051)*	− **0.1 (**− **0.192 to **− 0.017)*	0.0257 (− 0.063 to 0.102)
	High	Low	0.909 (0.828–0.997)	0.902 (0.817–0.996)	0.924 (0.832–1.026)	0.912 (0.828–1.005)	0.905 (0.816–1.005)
		High	0.961 (0.811–1.138)	0.972 (0.812–1.163)	0.936 (0.768–1.141)	1.002 (0.842–1.192)	0.913 (0.7501–1.112)
	RERI^b^		0.035 (0.006–0.08)*	0.059 (0.027–0.111)*	− 0.021 (− 0.05 to 0.031)*	**0.076 (0.042**–0.128)*	− 0.013 (− 0.0409 to 0.038)
Relatively humidity	Medium	Low	Reference layer	Reference layer	Reference layer	Reference layer	Reference layer
		High	1.023 (0.982–1.066)	1.028 (0.985–1.074)	1.011 (0.964–1.06)	1.021 (0.978–1.066)	1.025 (0.979–1.074)
	Low	Low	0.995 (0.903–1.097)	1.012 (0.912–1.122)	0.959 (0.857–1.074)	0.987 (0.89–1.094)	1.005 (0.901–1.121)
		High	1.023 (0.918–1.141)	1.022 (0.911–1.147)	1.026 (0.906–1.162)	1.004 (0.894–1.126)	1.045 (0.927–1.178)
	RERI^a^		0.005 (− 0.022 to 0.033)	− 0.018 (− 0.049 to 0.014)	0.056 (0.028–0.085)*	− 0.004 (− 0.036 to 0.026)	0.015 (− 0.017 to 0.047)
	High	Low	1.012 (0.905–1.131)	1.023 (0.909–1.152)	0.987 (0.868–1.123)	0.992 (0.881–1.117)	1.034 (0.913–1.171)
		High	0.919 (0.828–1.019)	0.904 (0.809–1.011)	0.952 (0.845–1.073)	0.925 (0.828–1.032)	0.912 (0.811–1.025)
	RERI^b^		− **0.115 (**− **0.176 to **− 0.058)*	− **0.147 (**− **0.214 to **− 0.084)*	− 0.043 (− 0.107 to 0.015)	− **0.088 (**− **0.151 to **− 0.031)*	− **0.144 (**− **0.216 to **− 0.079)*

Significant values are in bold.

*indicates P < 0.05; RERIa: meteorological factor low level-pollutant; RERIb: meteorological factor high level-pollutant.

The results of the bivariate response surface model (Fig. 4B) showed that the effects of ambient CO on ischemic stroke hospitalization increased with rising temperatures. Notably, the number of ischemic stroke hospitalizations peaked when both temperatures and CO levels were high. Synergistic effects of high temperature and CO on the risk of hospitalization in the total ischemic stroke population (*RERI* = 0.035, 95% CI 0.006–0.08) were noted. Subgroup analyses revealed synergistic effects of high temperature and CO on the risk of hospitalization in the young and middle-aged group (*RERI* = 0.059, 95% CI 0.027–0.111), and the male group (*RERI* = 0.076, 95% CI 0.042–0.128). Additionally, hypothermia and CO had antagonistic effects on the risk of ischemic stroke hospitalization in the elderly group (*RERI* = − 0.143, 95% CI − 0.25 to − 0.051) and in the male group (*RERI* = − 0.1, 95% CI − 0.192 to − 0.017). It’s important to note that high temperature, hypothermia, and CO had no interaction effects on the risk of ischemic stroke hospitalization in the female group.

### Interaction of CO and relative humidity on the risk of stroke hospitalization

**Figure 5:**
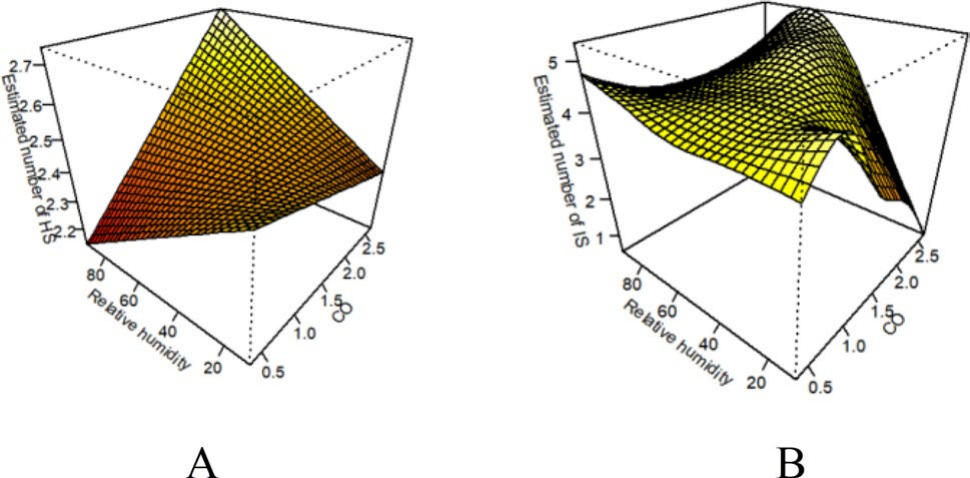
3D plot of the effect of ambient CO versus relative humidity on stroke.(**A** is a 3D plot of the effect of CO and relative humidity on hemorrhagic stroke. **B** is a 3D plot of the effect of CO and relative humidity on ischemic stroke.)

As shown in Fig. 5A and Table 3, low relative humidity and ambient CO had synergistic effects on the risk of hospitalization for the total population with hemorrhagic stroke (*RERI* = 0.192, 95% CI 0.184–0.205). Results from the subgroup analyses showed that low relative humidity and CO had synergistic effects on the risk of hospitalization for the male group (*RERI* = 0.175, the 95% CI 0.163–0.194), female group (*RERI* = 0.22, 95% CI 0.216–0.234), young and middle-aged group (*RERI* = 0.196, 95% CI 0.185–0.214), and elderly group (*RERI* = 0.1877, 95% CI 0.178–0.205). There was no interaction between high relative humidity and CO as far as the risk of hemorrhagic stroke hospitalization in any of the different subgroups was concerned.

As shown in Fig. 5B and Table 3, Low relative humidity did not interact with CO to affect the risk of hospitalization in the total population of ischemic stroke. On the other hand, high relative humidity and CO had antagonistic effects on the risk of hospitalization in the total population of ischemic stroke (*RERI* = − 0.115, 95% CI − 0.176 to − 0.058). In the age subgroup, low relative humidity and CO had synergistic effects on the risk of ischemic stroke hospitalization in the elderly group (*RERI* = 0.056, 95% CI 0.025–0.085) while high relative humidity and CO had antagonistic effects on the risk of ischemic stroke hospitalization in the young group (*RERI* = − 0.147, 95% CI − 0.214 to − 0.084). In the sex subgroup, high relative humidity and CO exhibited antagonistic effects on the risk of ischemic stroke hospitalization in both male and female groups (*RERI* = − 0.088, 95% CI − 0.151 to − 0.031; *RERI* = − 0.144, 95% CI − 0.216 to − 0.197. Low relative humidity did not have any notable interactions.

## Discussion

The results of this study suggest that there is a positive association between environmental CO and the risk of stroke hospitalization in the studied population. It was also noted that for hemorrhagic stroke, men and residents who were older than 65 years of age may be more sensitive to environmental CO, while there were no significant differences by age group with regard to ischemic stroke. In the gender group, women were more sensitive to environmental CO. Studies at home and abroad have shown that there is a positive association between stroke hospitalization and CO^20,21^, which is consistent with the results of this paper. A possible mechanism is that exposure to carbon monoxide impairs mitochondrial function in organisms, thereby generating mitochondrial reactive oxygen species, as well as releasing pro-inflammatory mediators and pro-apoptotic mediators. CO also modulates signaling pathways, thereby affecting key biological processes, including autophagy, mitochondrial biogenesis, programmed cell death (apoptosis), cell proliferation, as well as inflammation and innate immune response^22^. This, in turn, induces a number of cardiovascular diseases that lead to stroke.

Previous studies^23^ have shown that temperature is a major factor affecting the concentration of air pollutants.CO and high temperatures have synergistic effects on the risk of hospitalization for stroke. Previous systematic reviews and time-series studies have yielded similar results^24,25^. This may be due to the fact that sustained high temperatures promote photochemical reactions in the atmosphere, leading to an increase in the production of atmospheric pollutants such as CO. In addition, there is a common biological mechanism for the effects of temperature and atmospheric pollutants on population health, whereby extreme temperatures exacerbate inflammatory responses and trigger damage to the endothelium of the blood vessels, resulting in increased cholesterol levels and blood viscosity, as well as altered coagulation function. This ultimately leads to a range of human health effects^26,27^.

Changes in temperature are often accompanied by changes in humidity, and studies have shown that high and low relative humidity may be associated with the risk of stroke hospitalization^28^, this study found that high humidity has an antagonistic effect with CO, low humidity has a synergistic effect with CO, and higher humidity may help reduce pollutants in the air, including CO, because water vapor may promote the sedimentation of some pollutants. In addition, a high humidity environment may help maintain the body’s water balance and reduce the risk of dehydration, which may have a protective effect on the cardiovascular system. In a high humidity environment, the increased effect of CO on stroke risk may be offset or diminished by the potential benefits of humidity itself. Low relative humidity may mean less moisture in the air, which may exacerbate the dryness of the respiratory tract, which affects the respiratory system’s defense mechanisms. At the same time, carbon monoxide, as a colorless and odorless toxic gas, can spread and accumulate in the air more easily in low humidity environments, increasing the risk of human inhalation. In addition, Ningxia is located in the northwest of the province with a dry climate, and when the summer is hot, it will be accompanied by low relative humidity, so there is a synergistic effect between high temperature and low humidity and CO on the risk of stroke hospitalization.

The results from the subgroup analyses indicated that the effects of CO on the risk of hospitalization for stroke in the population were more sensitive among male than female, as it was among the elderly than the young and middle-aged, which is consistent with the findings of Liu et al.^21^. In addition, a recent study on cardiovascular disease (CVD) risk factors in China reported that male adults were associated with a relatively high prevalence of smoking, alcohol consumption, hypertension, and diabetes, which may make them more susceptible to the effects of air pollution. The stronger association of older adults with air pollution may be related to a gradual decrease in physiologic processes^20,29–32^, reduced clearance of air pollutants from the airways, and a higher prevalence of preexisting cardiovascular and respiratory diseases^33^. Therefore, more attention should be paid to the elderly due to their higher risk of exposure to air pollution.

This study had some limitations. First, we used the average of meteorological and air pollution data from five urban areas as an indicator of residential exposure, not taking into account the differences in CO concentrations, temperatures, and relative humidity in different areas. This could lead to misclassification of exposures and a potential bias in the study results. Second, the limited availability of data made it difficult to control more detailed meteorological variables (e.g. wind speed and barometric pressure), demographic information (e.g. education level and socioeconomic status), and other relevant behavioral risk factors (e.g. smoking status, physical activity, and dietary habits). In addition, we obtained the dates of hospitalization for stroke rather than the onset dates, which in some stroke patients may have been a few days prior to admission. Furthermore, we did not consider cases where death occurred prior to admission, and such uncertainties may have led to information bias in the exposure assessment. However, stroke is an acute disease with severe symptoms, and in China, most stroke patients are likely to visit the nearest hospital within six hours after the onset of symptoms^34^, so misclassification bias may be limited. Finally, the data were collected in one province in northwest China, which may limit the generalizability of the findings from this study. Therefore, further large-scale studies in developing countries are encouraged to validate the findings from the current study in different populations.

## Conclusion

Short-term exposure to CO increases the risk of hospitalization for both hemorrhagic and ischemic stroke in the population. The interactions between CO and meteorological factors had effects on the risk of hospitalization for stroke. The effects tend to vary with age and gender differences. Therefore, care should be taken to protect susceptible populations under specific climatic conditions to reduce their risk of being affected by hemorrhagic or ischemic stroke.

## Data Availability

The datasets generated during and/or analysed during the current study are available from the corresponding author on reasonable request.
